# Phenotypic variation in biomass and related traits among four generations advanced lines of Cleome (*Gynandropsis gynandra* L. (Briq.))

**DOI:** 10.1371/journal.pone.0275829

**Published:** 2022-10-12

**Authors:** Aristide Carlos Houdegbe, Enoch G. Achigan-Dako, E. O. Dêêdi Sogbohossou, M. Eric Schranz, Alfred O. Odindo, Julia Sibiya

**Affiliations:** 1 School of Agricultural, Earth and Environmental Sciences, University of KwaZulu-Natal, Pietermaritzburg, Republic of South Africa; 2 Faculty of Agronomic Sciences, Laboratory of Genetics, Biotechnology and Seed Science, University of Abomey-Calavi, Abomey-Calavi, Republic of Benin; 3 Biosystematics Group, Wageningen University, Wageningen, The Netherlands; KGUT: Graduate University of Advanced Technology, ISLAMIC REPUBLIC OF IRAN

## Abstract

*Gynandropsis gynandra* (spider plant) is an African traditional leafy vegetable rich in minerals, vitamins and health-promoting compounds with potential for health promotion, micronutrients supplementation and income generation for stakeholders, including pharmaceutical companies. However, information on biomass productivity is limited and consequently constrains breeders’ ability to select high-yielding genotypes and end-users to make decisions on suitable cultivation and production systems. This study aimed to assess the phenotypic variability in biomass and related traits in a collection of *G*. *gynandra* advanced lines to select elite genotypes for improved cultivar development. Seventy-one advanced lines selected from accessions originating from Asia, West Africa, East Africa and Southern Africa were evaluated over two years with two replicates in a greenhouse using a 9 x 8 alpha lattice design. Significant statistical differences were observed among lines and genotype origins for all fourteen biomass and related traits. The results revealed three clusters, with each cluster dominated by lines derived from accessions from Asia (Cluster 1), West Africa (Cluster 2), and East/Southern Africa (Cluster 3). The West African and East/Southern African groups were comparable in biomass productivity and superior to the Asian group. Specifically, the West African group had a low number of long primary branches, high dry matter content and flowered early. The East/Southern African group was characterized by broad leaves, late flowering, a high number of short primary branches and medium dry matter content and was a candidate for cultivar release. The maintenance of lines’ membership to their group of origin strengthens the hypothesis of geographical signature in cleome diversity and genetic driver of the observed variation. High genetic variance, broad-sense heritability and genetic gains showed the potential to improve biomass yield and related traits. Significant and positive correlations among biomass per plant, plant height, stem diameter and leaf size showed the potential of simultaneous and direct selection for farmers’ desired traits. The present results provide insights into the diversity of spider plant genotypes for biomass productivity and represent key resources for further improvement in the species.

## Introduction

*Gynandropsis gynandra* (L.) Briq. (Syn. *Cleome gynandra* L.), commonly known as spider plant, is an African leafy vegetable with great potential in addressing micronutrient deficiency [1], which affects more than two billion people worldwide, mainly in Asia and sub-Saharan Africa [2]. The leaves of the species are rich in vitamins C, A, E, B1, B2, and B9 and minerals such as iron, zinc, calcium, copper, potassium, magnesium, manganese, phosphorus and sodium [1,3–6]. *Gynandropsis gynandra* leaves are also an important source of proteins and fatty acids [7,8], including essential amino acids (histidine, isoleusine, leucine, lysine, methionine, phenylalanine, threonine, valine) [7]. In addition, spider plant has several health-promoting properties, as it contains numerous secondary metabolites, such as flavonoids, terpenoids, tannins, glucosinolates, aldehydes, ketones, sesquiterpenes and many other phenolic compounds [1,4,9–12] with diverse pharmaceutical applications (plant extracts, drugs, etc.) [13]. The species is a prime resource for the pharmaceutical industry, as its extracts have several biological and pharmacological effects [1,13–15], including antimicrobial (fungi and bacteria), anthelmintic [16], antimalarial [17], hepatoprotective [18], antiarthritic [19], antioxidant, anti-inflammatory [20], immunomodulatory [21], antinociceptive [22], anticancer [23], antidiabetic [24] and vasodilatory [25] activities. Promoting this vegetable will, therefore, contribute to fighting malnutrition, health promotion and income generation for stakeholders, including pharmaceutical companies and local communities.

*Gynandropsis gynandra* belongs to the Cleomaceae family and is found in tropical and subtropical areas across all continents but is used mainly by local communities in Africa and Asia [3,26,27]. The leaves, young and tender shoots and flowers are used to prepare stews and sauces which are eaten as vegetables. The species is also used in traditional medicine. For instance, Sogbohossou et al. [28] reported its utilization in curing more than 40 diseases in Togo and Benin and various uses among Ewe, Adja, Fon, Holli, Waama, Gourmantche, and Zerma socioethnic groups. These uses in local pharmacopoeia together with the scientific evidence of large variation in health promoting compounds, support the development of cleome extracts and drugs [13]. *Gynandropsis gynandra* is a semicultivated crop mainly found near human settlements, along roadsides, irrigation canals and ditches, and cultivated fields or fallows as wild populations [29,30]. The species is cultivated in home gardens and peri-urban and urban market gardening across sub-Saharan Africa [28,31,32]. Spider plant is grown mainly as monoculture in rainy seasons but also throughout the year when irrigation is available. The crop does not tolerate temperature below 15°C and is sensitive to cold [33,34]. For example, the recommended growing period include spring (September to November) and summer (December to March) in South Africa [34]. Direct seeding as well as transplanting are all practiced. The cultivation and commercialisation of the species are mainly done by women and provide substantial income for households [28,35,36]. The leaves are sold in open markets in many African countries (e.g., Kenya, Namibia, Benin, Tanzania, South Africa, Togo, Ghana, Burkina-Faso, Uganda) but also in supermarkets (e.g., Kenya) [29,32,35,36]. The species production from home gardens generated a profit margin between 40% to 57% and production efficiency (benefit cost ratio) between 1.66 and 2.33 during the rainy season in the Adja community of Benin [35]. The demand for spider consumption is increasing across sub-Saharan African countries. For instance, spider plant production increased from 19 428 metric tons to 21 507 metric tons between 2012 and 2013, with an average increase of 50% in cultivated area in Kenya [37].

In areas where *G*. *gynandra* is cultivated, production constraints faced by farmers include poor germination, early flowering, low yield, insect pests and seed availability [35,36,38]. Studies addressing these constraints reported that dormancy was responsible for erratic germination, and treating seeds with gibberellic acid and preheating were found to be effective [39–41], as well as storage for three or more months [42]. Improving leaf yield, early flowering, and insect pest resistance can be achieved by developing improved agricultural practices and high-yielding cultivars. Most previous studies focused on establishing the best agronomic practices for improved yield and included optimal planting density, type and fertilizer application rates, planting date, stage of transplanting, harvesting frequency and techniques (cutting, uprooting whole plants, defoliation), deflowering, sowing depth and net cover colour [43–49]. In contrast, limited studies thus far have addressed the genetic improvement of the species [13].

Genetic improvement requires a better understanding of the genetic diversity in the species through morphological and genetic/genomic characterization. Many studies have assessed morphological diversity in *G*. *gynandra* using a countrywide collection (e.g., Ghana [50], Burkina-Faso [51], Kenya [52,53]), regionwide germplasm (e.g., Kenya and South Africa [54], East and Southern Africa [4]) and worldwide collection [3,55]. It is worthwhile to highlight that some of these characterization studies were extended to nutritional values, including minerals [4], vitamins [3], and physiological traits [56]. Significant variations were observed among accessions with a strong association between their morphology and geographical origins [3,55]. East-Southern African accessions were observed to have taller plants compared to Asian and West African accessions with shorter plants [3]. Additionally, West African accessions were characterized by small leaves, and Asian and East-Southern African accessions had large leaves [3]. This morphological differentiation was further supported by genomic characterization [57]. Genetic differentiation was also observed between farmer’s cultivars and genebank’s accessions and advanced lines [58]. The considerable diversity observed represents a valuable resource for a successful breeding program.

However, most studies assessing morphological diversity in *G*. *gynandra* did not include leaf biomass yield. Those that included it were limited to regional accessions and advanced lines [4] and countrywide accessions [51,53]. Whereas farmers prefer traits in *G*. *gynandra* that include high leaf yield and related traits (plant height and the number of leaves), broad leaves, late flowering, good germination and resistance to pests and diseases [32,59–61]. Among these traits, yield is the most important trait for farmers and breeding programs. Considering farmers’ preferred traits in a breeding program is vital in the successful adoption of developed cultivars. Given the availability of worldwide collections, it is, therefore, important to assess the biomass potential of large germplasm collections.

Spider plant is both self- and cross-compatible but predominantly out-crossing [58,62,63], opening the rooms for developing both inbred/pure lines and hybrid cultivars. The outcrossing was observed to be exacerbated by the crop flowers’ visits by both diurnal insect pollinators (bees, ants and butterfly) [33,62,63], and nocturnal pollinators (e.g. *Hippotion* spp, and *Nephele aequivalens*) [64] due to its flower structure. Spider plant has three types of flowers: staminate with short gynoecium, hermaphrodite with medium gynoecium, and hermaphrodite with long gynoecium, characteristics of an andromonoecious plant [63]. Giving the predominance of out-crossing in the species, hybrids cultivars could be advantageous over inbred lines through the exploitation of heterosis. In hybrid production, an important step is to develop inbred/pure lines. Various methods (e.g. single seed descent, bulk, pedigree, and doubled-haploids) can be used in developing inbred lines and single seed descend (SSD) has the advantage to allow a rapid development of inbred lines in a greenhouse or off-season [65].

Therefore, this study aimed to assess the phenotypic diversity in biomass yield and related traits among a worldwide collection of *Gynandropsis gynandra* advanced lines developed from SSD method to select elite genotypes for breeding programs and large-scale dissemination. Specifically, the present study: (i) assessed the phenotypic variation in biomass and related traits in *G*. *gynandra* using advanced lines selected from Asian, West, East and Southern African accessions; (ii) determined the relationship between biomass yield and related traits; and (iii) identified the best-performing genotypes for biomass yield.

## Materials and methods

### Plant material

In this study, seventy-one advanced lines (Table 1) selected from accessions originating from Asia (18), West Africa (19), Eastern Africa (14) and Southern Africa (20) were evaluated. The accessions were obtained from the Laboratory of Genetics, Biotechnology and Seed Science of the University of Abomey-Calavi (Republic of Benin); the World Vegetable Center (Taiwan); the Kenya Resource Center for Indigenous Knowledge (Kenya); the Lilongwe University of Agriculture and Natural Resources (Malawi); the Namibia Botanical Gardens (Namibia); the Wageningen University and Research (Netherlands) and the University of Ouagadougou (Burkina-Faso) (Table 1). Accessions were self-pollinated for four generations to develop the advanced lines using a single seed descent method. Briefly, only one seed was picked from each selfed plant per original accession. The single seed was then planted in the next generation of selfing, and the procedure was repeated until the fourth generation. Seeds of the fourth selfing generation pods were bulked for evaluation.

**Table 1:** List of advanced lines of *Gynandropsis gynandra* used in this study and their origin.

Genotype	Genebank holding of the original accession	Country of Origin	Region
**EA1**	National Museums of Kenya	Kenya	East Africa
**EA2**	National Museums of Kenya	Kenya	East Africa
**EA3**	National Museums of Kenya	Kenya	East Africa
**EA4**	National Museums of Kenya	Kenya	East Africa
**WA1**	University of Ouagadougou	Burkina-Faso	West Africa
**WA2**	University of Ouagadougou	Burkina-Faso	West Africa
**EA5**	National Museums of Kenya	Kenya	East Africa
**EA6**	National Museums of Kenya	Kenya	East Africa
**WA3**	University of Ouagadougou	Burkina-Faso	West Africa
**WA4**	Laboratory of Genetics, Biotechnology and Seed Science (GBioS), University of Abomey-Calavi	Benin	West Africa
**WA5**	Laboratory of Genetics, Biotechnology and Seed Science (GBioS), University of Abomey-Calavi	Benin	West Africa
**WA6**	Laboratory of Genetics, Biotechnology and Seed Science (GBioS), University of Abomey-Calavi	Benin	West Africa
**WA7**	Laboratory of Genetics, Biotechnology and Seed Science (GBioS), University of Abomey-Calavi	Benin	West Africa
**WA8**	Laboratory of Genetics, Biotechnology and Seed Science (GBioS), University of Abomey-Calavi	Benin	West Africa
**WA9**	Laboratory of Genetics, Biotechnology and Seed Science (GBioS), University of Abomey-Calavi	Benin	West Africa
**WA10**	Laboratory of Genetics, Biotechnology and Seed Science (GBioS), University of Abomey-Calavi	Benin	West Africa
**WA11**	Laboratory of Genetics, Biotechnology and Seed Science (GBioS), University of Abomey-Calavi	Togo	West Africa
**WA12**	Laboratory of Genetics, Biotechnology and Seed Science (GBioS), University of Abomey-Calavi	Togo	West Africa
**WA13**	Laboratory of Genetics, Biotechnology and Seed Science (GBioS), University of Abomey-Calavi	Togo	West Africa
**WA14**	Laboratory of Genetics, Biotechnology and Seed Science (GBioS), University of Abomey-Calavi	Togo	West Africa
**WA15**	Laboratory of Genetics, Biotechnology and Seed Science (GBioS), University of Abomey-Calavi	Togo	West Africa
**WA16**	Laboratory of Genetics, Biotechnology and Seed Science (GBioS), University of Abomey-Calavi	Togo	West Africa
**WA17**	Laboratory of Genetics, Biotechnology and Seed Science (GBioS), University of Abomey-Calavi	Togo	West Africa
**WA18**	Laboratory of Genetics, Biotechnology and Seed Science (GBioS), University of Abomey-Calavi	Togo	West Africa
**WA19**	Laboratory of Genetics, Biotechnology and Seed Science (GBioS), University of Abomey-Calavi	Ghana	West Africa
**AS1**	World Vegetable Center	Thailand	Asia
**AS2**	World Vegetable Center	Lao People’s Democratic Republic	Asia
**AS3**	World Vegetable Center	Lao People’s Democratic Republic	Asia
**AS4**	World Vegetable Center	Lao People’s Democratic Republic	Asia
**AS5**	World Vegetable Center	Thailand	Asia
**AS6**	World Vegetable Center	Thailand	Asia
**EA7**	World Vegetable Center	Kenya	East Africa
**SA1**	World Vegetable Center	Zambia	Southern Africa
**AS7**	World Vegetable Center	Lao People’s Democratic Republic	Asia
**AS8**	World Vegetable Center	Malaysia	Asia
**AS9**	World Vegetable Center	Malaysia	Asia
**AS10**	World Vegetable Center	Malaysia	Asia
**AS11**	World Vegetable Center	Malaysia	Asia
**AS12**	World Vegetable Center	Lao People’s Democratic Republic	Asia
**EA8**	World Vegetable Center	Uganda	East Africa
**EA9**	World Vegetable Center	Uganda	East Africa
**EA10**	World Vegetable Center	Uganda	East Africa
**EA11**	World Vegetable Center	Uganda	East Africa
**SA2**	World Vegetable Center	Malawi	Southern Africa
**SA3**	World Vegetable Center	Malawi	Southern Africa
**EA12**	World Vegetable Center	Kenya	East Africa
**EA13**	World Vegetable Center	Kenya	East Africa
**SA4**	World Vegetable Center	South Africa	Southern Africa
**SA5**	World Vegetable Center	Zambia	Southern Africa
**AS13**	World Vegetable Center	Taiwan	Asia
**SA6** *	Laboratory of Genetics, Biotechnology and Seed Science (GBioS), University of Abomey-Calavi	Mozambique	Southern Africa
**EA14**	National Museums of Kenya	Kenya	East Africa
**AS14**	World Vegetable Center	Malaysia	Asia
**AS15**	World Vegetable Center	Thailand	Asia
**AS16**	World Vegetable Center	Lao People’s Democratic Republic	Asia
**AS17**	World Vegetable Center	Lao People’s Democratic Republic	Asia
**SA7**	Okakarara	Namibia	Southern Africa
**SA8**	Otjiwarongo	Namibia	Southern Africa
**SA9**	Lilongwe University of Agriculture and Natural Resources	Malawi	Southern Africa
**SA10**	Lilongwe University of Agriculture and Natural Resources	Malawi	Southern Africa
**SA11**	Mahenene Research Station	Namibia	Southern Africa
**SA12**	Chitedze Research Station	Malawi	Southern Africa
**SA13**	Namibia Botanical Gardens	Namibia	Southern Africa
**SA14**	Namibia Botanical Gardens	Namibia	Southern Africa
**SA16** *	Laboratory of Genetics, Biotechnology and Seed Science (GBioS), University of Abomey-Calavi	Zimbabwe	Southern Africa
**AS18**	Wageningen University and Research	Malaysia	Asia
**SA17**	Okakarara	Namibia	Southern Africa
**SA18**	Lilongwe University of Agriculture and Natural Resources	Malawi	Southern Africa
**SA19**	Lilongwe University of Agriculture and Natural Resources	Malawi	Southern Africa
**SA20**	Chitedze Research Station	Malawi	Southern Africa
**SA21** *	Laboratory of Genetics, Biotechnology and Seed Science (GBioS), University of Abomey-Calavi	Zimbabwe	Southern Africa

*, Provided to the Laboratory of Genetics, Biotechnology and Seed Science (GBioS) of University of Abomey-Calavi by Mr Tomas Massingue (Mozambique) and Dr Admire Shayanowako (Zimbabwe).

### Experimental design and growth conditions

The advanced lines were evaluated in 2020 (September to December) and 2021 (January to April) under greenhouse conditions at the Controlled Environment Facility (29°46′ S, 30°58′ E) of the University of KwaZulu-Natal, Pietermaritzburg Campus, South Africa. Each year, the evaluation was laid out in a 9 x 8 alpha design with two replications. Seeds were pretreated by heating at 40°C for three days to improve germination before sowing in seedling trays filled with growing media. The seedling trays were established in the greenhouse, and germination was observed three days after planting. Seedlings were grown for four weeks in a nursery and transplanted in 10 litre pots with three plants per pot. Pots were filled with composted pine bark growing media. Basal fertilizer composed of N:P:K (2:3:2) at a dose of 150 kg ha^-1^ was applied before transplanting, and limestone ammonium nitrate (28% N) was applied as topdressing two weeks after transplanting at a dose of 100 kg ha^-1^. Automated drip irrigation was used to water the plants with 1 litre per pot daily, while weeds were controlled manually. In 2020, the average temperature and relative humidity were 28°C day/20°C night and 78.5%, respectively. The average temperature and relative humidity were 31°C day/22°C night and 77.4%, respectively, in 2021.

### Data collection

Fourteen agronomic traits, including days to 50% flowering (DFlow), stem diameter (StDiam), plant height (PHeight), number of primary branches (NPBr), primary branch length (PBrLeng), central leaflet length (CtLleng), central leaflet width (CtLwid), leaf width (Lwid), petiole length (Ptilleng), leaf area (LfArea), total fresh biomass per plant (FBiom), edible fresh biomass per plant (EDBiom), harvest index (HI) and dry matter content (DM), were assessed four weeks after transplanting. Days to 50% flowering were recorded as the number of days from the sowing date to the day when 50% of the plants in each pot flowered. The central leaflet length (cm), central leaflet width (cm), leaf width (cm) and petiole length (cm) were collected on a fully developed primary leaf randomly selected on each plant using a ruler. The selected leaf was scanned using a Canon PIXMA G2411 scanner (Canon INC; Tokyo, Japan), and the resultant image was used to calculate leaf area using the R package “*LeafArea*” [66]. Plant height (cm) was measured from the base to the top of the plant with a tape measure, while the stem diameter was measured using a digital Vernier calliper at the plant collar. Each plant was harvested by cutting at a height of 15 cm above the ground, and the resultant biomass was weighed to determine the total fresh biomass per plant (g plant^-1^). The edible part of the total biomass was separated and weighed to record the edible fresh biomass per plant (g plant^-1^). The ratio of edible biomass to total fresh biomass was computed and reported as the harvest index (HI). All phenotypic traits measurements were taken on two plants out of the three plants per pot, except days to 50% flowering and dry matter content. An average value from the two individual plants per pot was computed and used in the data analysis. For dry matter content (DM), edible biomass of the plants per genotype in each replicate was bulked, and a sample of 20 g was taken and oven-dried at 65°C for 72 h. DM (%) was computed as DM = (dry weight)/(fresh weight) x 100. The phenotypic data are presented in S1 Table.

### Data analysis

The quality of data was assessed for outlier detection following Bernal-Vasquez et al. [67] using the Bonferroni–Holm test based on studentized residuals at the significance level of 5%. The mean, minimum, maximum, coefficient of variation and standard deviation were generated to characterize the plant material using the function *describe* of the R package *“psych”* [68]. The difference among regions of origin was tested using an analysis of variance or Kruskal–Wallis test, when necessary. Data were first analyzed separately per year by fitting a linear mixed model according to the following statistical model:

$$y_{ikl}=\mu +R_{k}+B_{l}(R_{k})+G_{i}+\varepsilon_{ikl}$$ (1)

in which *y*_*ikl*_ was the phenotypic observation of the *i*^*th*^ line in the *l*^*th*^ incomplete block within the *k*^*th*^ replicate, *μ* was the overall mean, *B*_*l*_(*R*_*k*_) was the random effect of the *l*^*th*^ incomplete block within the *k*^*th*^ replicate, *G*_*i*_ was the random effect of the *i*^*th*^ line, and *ε*_*ikl*_ was the random residual.

Variance components across years were estimated by fitting a linear mixed-effect model using the restricted maximum likelihood (REML) implemented in the ASReml-R package version 4.1.0.160 [69] according to the following statistical model:

$$y_{ijkl}=\mu +Y_{j}+R_{k}(Y_{j})+B_{l}[R_{k}(Y_{j})]+G_{i}+{GY}_{ij}+\varepsilon_{ijkl}$$ (2)

in which *y*_*ijkl*_ was the phenotypic observation of the *i*^*th*^ line in the *l*^*th*^ incomplete block within the *k*^*th*^ replicate at the *j*^*th*^ year, *μ* was the overall mean, *Y*_*j*_ was the random effect of the *j*^*th*^ year, *R*_*k*_(*Y*_*j*_) was the random effect of the *k*^*th*^ replicate within the *j*^*th*^ year, *B*_*l*_[*R*_*k*_(*Y*_*j*_)] was the random effect of the *l*^*th*^ incomplete block within the *k*^*th*^ replicate at the *j*^*th*^ year, *G*_*i*_ was the random effect of the *i*^*th*^ line, *GY*_*ij*_ was the random effect of the interaction between the *i*^*th*^ line and the *j*^*th*^ year, and *ε*_*ijkl*_ was the random residual. Heterogeneous variances were assumed for residual effects in different years. The likelihood ratio test [70] was used to test the significance of the variance components for single year and across years analyses using the function *lrt* implemented in the ASREML-R package. Standard broad-sense heritability across years [71] was calculated as follows:

$$H^{2}=\frac{\sigma_{G}^{2}}{\sigma_{G}^{2}+\frac{\sigma_{G\times Y}^{2}}{n}+\frac{\sigma_{e}^{2}}{nr}}$$ (3)

where $\sigma_{G}^{2}$ is the genotypic variance of the lines, $\sigma_{G\times Y}^{2}$ is the line × year interaction variance, $\sigma_{e}^{2}$ is the residual variance, *r* is the number of replications, and *n* is the number of years.

The phenotypic best linear unbiased predictors (BLUPs) were generated from model 2. BLUPs were used because they have good predictive accuracy over the best linear unbiased estimators (BLUEs) due to their high correlation with the true values and their ability to handle environmental effects and have been recommended for phenotypic selection in plant breeding [72–74]. The values refer to mean genotypic values and were used in further analyses. Pearson’s correlation coefficients among all traits and their level of significance were calculated using the function *corr* from the R package *“Hmisc”* [75]. Genotypic correlations among traits were estimated using META-R software [76]. Both genotypic and phenotypic correlations were plotted using the *“metan”* R package [77]. A principal component analysis was performed using the *PCA* function implemented in the R *“FactoMineR”* package [78] to assess the relationship among the lines and the biomass and related traits. Furthermore, we performed hierarchical clustering on principal components (HCPC) to group the genotypes based on the measured traits, and the results were visualized using the *fviz_cluster* and *fviz_dendogram* functions of the R package *“factoextra”* [79] for factor map and dendrogram, respectively. The significant difference among the means of the clusters was tested using one-way analysis of variance according to the following statistical model:

$$y_{mi}=\mu +C_{m}+\varepsilon_{mi}$$ (4)

in which *y*_*mi*_ was the phenotypic observation of the *i*^*th*^ line on the *m*^*th*^ cluster, *μ* was the overall mean, *C*_*m*_ was the fixed effect of the *m*^*th*^ cluster, and *ε*_*mi*_ was the random residual. In addition, the means of clusters were separated using Tukey’s honestly significant difference test (Tukey’s HSD post hoc test) at the 0.05 probability level using the R package “*agricolae*” [80].

The genetic advance (GA) for each trait was computed as *GA = i × H*^*2*^ *× σ*_*P*_, where *σ*_*P*_ was the phenotypic standard deviation, *H*^*2*^ was the broad-sense heritability, and *i* was the standardized selection differential at the selection intensity of 5% (i = 2.06) [81]. Genetic advance over mean (GAM) was further computed as *GAM = (GA/μ) × 100*, where *μ* was the overall mean and *GA* was the genetic advance of the trait. Genotypic, phenotypic and error coefficients of variation (GCV, PCV and ECV, respectively) were estimated according to Burton and DeVane [82] as follows:

$$GCV\;(\%)=\frac{\sqrt{\sigma_{G}^{2}}}{\mu }\times 100$$ (5)

$$PCV\;(\%)=\frac{\sqrt{\sigma_{P}^{2}}}{\mu }\times 100$$ (6)

$$ECV\;(\%)=\frac{\sqrt{\sigma_{e}^{2}}}{\mu }\times 100$$ (7)

in which $\sigma_{G}^{2}$ was the genotypic variance, $\sigma_{P}^{2}$ was the phenotypic variance, $\sigma_{e}^{2}$ was the residual variance, and *μ* was the overall mean. R software version 4.1.1 [83] was used to perform all statistical analyses.

## Results

### Quantitative variation in biomass and related traits

A highly significant variation (p < 0.001) was observed among genotypes for all agronomic traits each and across years (Tables 2 and 3). Blocks did not significantly affect all the agronomic traits within and across years except days to 50% flowering. Similarly, year effects were not significant for all traits except stem diameter and days to 50% flowering. Replicates effects were significant only for plant height, leaf width (in 2020 or 2021 and across years), dry matter content (in 2021 and across years) and central leaflet width (across years). The genotype × year interaction effects were significant for stem diameter, primary branch length, number of primary branches, leaf width and area, petiole length, harvest index and days to 50% flowering (Table 3).

**Table 2:** Descriptive statistics of biomass and related traits investigated in 71 advanced lines of *Gynandropsis gynandra*.

Traits	Mean	Minimum	Maximum	Standard deviation	Coefficient of variation (%)
**StDiam: stem diameter (mm)**	9.94	2.27	18.72	2.85	28.63
**PHeight: plant height (cm)**	70.6	13	117.5	20.99	29.74
**NPBr: number of primary branches**	10.7	2.5	23.50	4.19	39.19
**PBrLeng: primary branch length (cm)**	31.04	0.2	106	25.6	82.48
**CtLleng: central leaflet length (cm)**	7.17	2.5	12.35	1.77	24.64
**CtLwid: central leaflet width (cm)**	3.17	1	5.50	0.71	22.43
**Lwid: leaf width (cm)**	10.9	4	19.60	3.14	28.78
**Ptillen: petiole length (cm)**	10.95	4.50	20.35	3.11	28.39
**LfArea: leaf area (cm** ^**2**^ **)**	53.22	5.64	147.76	26.59	49.97
**FBiom: total fresh biomass per plant (g)**	67.19	2.10	248.40	42.73	63.59
**EDBiom: edible fresh biomass per plant (g)**	28.34	1.20	101.90	17.31	61.08
**HI: harvest index**	0.47	0.24	0.91	0.12	25.78
**DM: dry matter content (%)**	10.67	7.60	15.42	1.5	14.01
**DFlow: days to 50% flowering (days)**	60.14	32	95	13.88	23.07

**Table 3:** Likelihood ratio test (LRT) for replicate, block, genotype, and genotype × year interaction effects regarding fourteen agronomic traits in 2020 and 2021, and across years for 71 advanced lines of *Gynandropsis gynandra*.

Traits	Years	Replicate	Block (replicate)	Genotype	Year	Genotype × Year
**StDiam**	2020	0.003ns	0.000ns	40.494***	-	-
	2021	0.000ns	0.000ns	19.39***	-	-
	Across years	0.000ns	0.000ns	24.844***	3.232*	3.835*
**PHeight**	2020	5.599**	2.266ns	35.556***	-	-
	2021	0.138ns	0.000ns	18.013***	-	-
	Across years	9.178**	0.16ns	25.002***	0.132ns	1.029ns
**PBrLeng**	2020	0.003ns	0.000ns	40.494***	-	-
	2021	0.000ns	0.000ns	19.39***	-	-
	Across years	0.079ns	0.000ns	34.136***	0.000ns	4.749*
**NPBr**	2020	0.000ns	0.099ns	63.363***	-	-
	2021	0.22ns	1.003ns	30.168***	-	-
	Across years	0.000ns	0.000ns	49.354***	0.000ns	3.462*
**CtLleng**	2020	2.423ns	0.914ns	54.021***	-	-
	2021	0.403ns	0.000ns	20.221***	-	-
	Across years	2.909*	0.787ns	38.635***	0.021ns	1.34ns
**CtLwid**	2020	0.000ns	0.053ns	31.407***	-	-
	2021	0.000ns	0.000ns	22.616***	-	-
	Across years	0.000ns	0.146ns	30.861***	0.009ns	0.699ns
**Lwid**	2020	7.792**	0.001ns	62.488***	-	-
	2021	0.738ns	0.000ns	29.455***	-	-
	Across years	9.228**	0.000ns	32.418***	0.000ns	8.104**
**Ptillen**	2020	0.000ns	0.000ns	41.168***	-	-
	2021	0.000ns	1.251ns	58.655***	-	-
	Across years	0.000ns	0.911ns	54.393***	0.000ns	5.716**
**LfArea**	2020	0.932ns	0.000ns	79.115***	-	-
	2021	1.505ns	0.000ns	31.052***	-	-
	Across years	1.76ns	0.000ns	35.542***	0.000ns	6.999**
**FBiom**	2020	0.122ns	1.834ns	37.035***	-	-
	2021	0.000ns	0.000ns	17.312***	-	-
	Across years	0.034ns	0.726ns	19.444***	0.000ns	1.577ns
**EDBiom**	2020	0.000ns	0.000ns	30.317***	-	-
	2021	0.000ns	0.000ns	16.546***	-	-
	Across years	0.000ns	0.000ns	15.857***	1.279ns	1.223ns
**HI**	2020	0.000ns	1.284ns	30.725***	-	-
	2021	1.371ns	0.41ns	17.828***	-	-
	Across years	0.62ns	2.686ns	17.049***	2.076ns	7.63**
**DM**	2020	0.05ns	0.169ns	19.433***	-	-
	2021	4.263*	0.954ns	8.549**	-	-
	Across years	5.068*	0.341ns	17.191***	0.339ns	1.675ns
**DFlow**	2020	0.000ns	2.434ns	64.399***	-	-
	2021	0.226ns	0.002ns	27.865***	-	-
	Across years	0.007ns	3.372*	21.382***	9.044**	31.41***

StDiam: Stem diameter (mm), PHeight: Plant height (cm), PBrLeng: Primary branch length (cm), NPBr: Number of primary branches, CtLleng: Central leaflet length (cm), CtLwid: Central leaflet width (cm), Lwid: Leaf width (cm), Ptillen: Petiole length (cm), LfArea: Leaf area (cm^2^), FBiom: Total fresh biomass per plant (g), EDBiom: Edible fresh, biomass per plant (g), HI: Harvest index, DM: Dry matter content (%), DFlow: Days to 50% flowering (days).

***, **, *: Significantly different from zero at the 0.001, 0.01, and 0.05 probability level, respectively. ns: Not significantly different from zero at the 0.05 level of probability.

The coefficient of variation evolved between 14.01% and 82.48%. Overall, lower values for dry matter content and higher values for primary branch length were observed. The average plant total fresh biomass and edible fresh biomass were 67.19 ± 2.67 g and 28.34 ± 1.08 g, respectively. As the second most variable trait, the plant total fresh biomass (CV = 63.59%) ranged from 2.10 g to 248.40 g, while the edible fresh biomass (CV = 61.08%) ranged between 1.20 g and 101.90 g per plant. The harvest index was 0.47 ± 0.01 on average with a range of 0.24–0.91. The spider plant genotypes flowered on average 60.14 ± 0.90 days after sowing, and days to 50% flowering ranged between 32 and 95 days after sowing. The plant height ranged from 13 cm to 117.5 cm, with an average of 70.6 ± 1.31 cm. The average number of primary branches was 10.7 ± 0.26 per plant and varied between 2.5 and 23.5. The single leaf area ranged from 5.64 to 147.76 cm^2^ with an average of 53.22 ± 1.66 cm^2^. The dry matter content was 10.67 ± 0.09% on average, with a range of 7.60–15.42.

**Fig 1:**
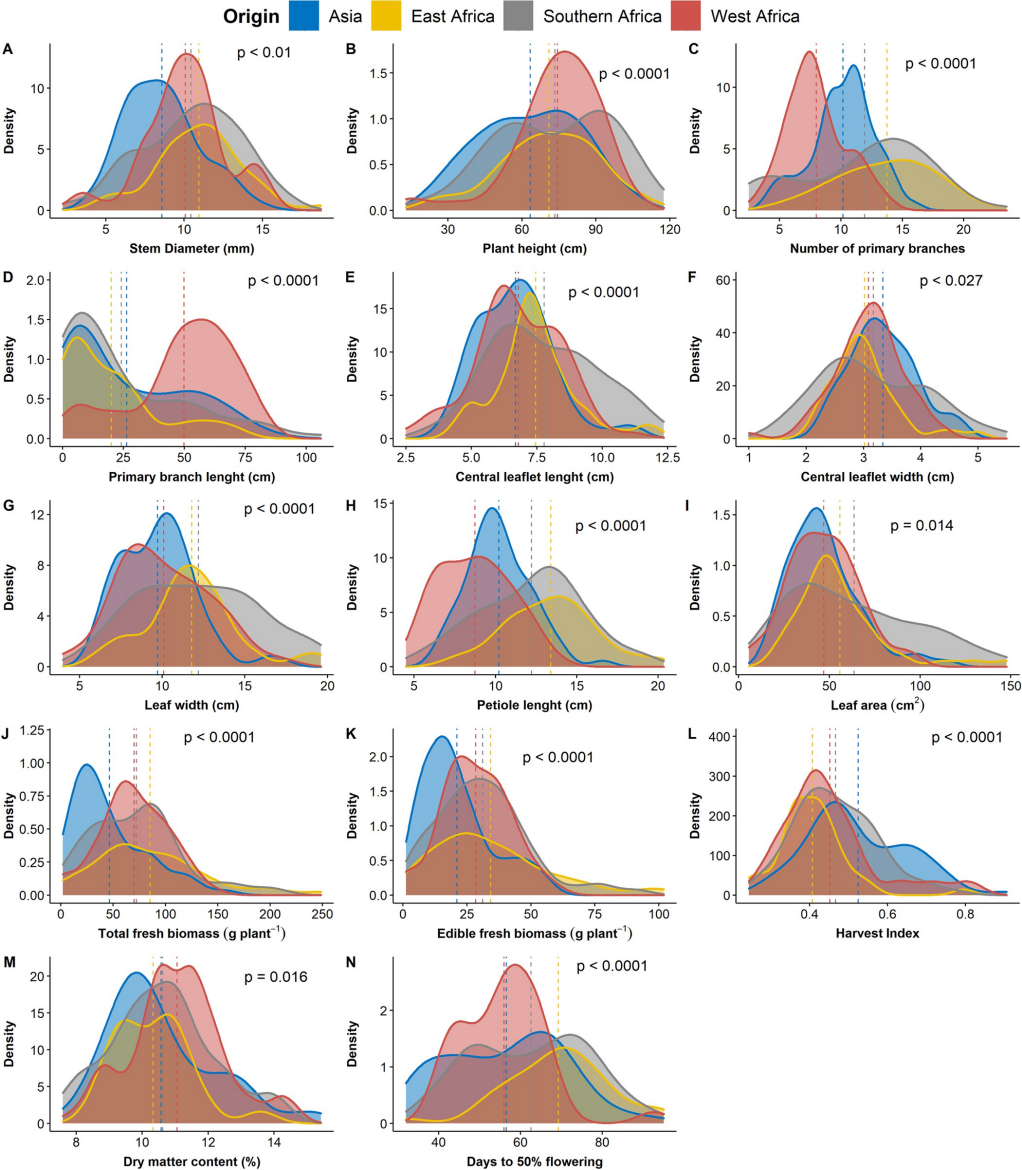
Distribution of phenotypic values for fourteen agronomic traits among regions of origin of 71 advanced lines of *Gynandropsis gynandra*. (A) stem diameter (mm). (B) Plant height (cm). (C) Primary branch length (cm). (D) Number of primary branches. (E) Central leaflet length (cm). (F) Central leaflet width (cm). (G) Leaf width (cm). (H) Petiole length (cm). (I) Leaf area (cm^2^). (J) Total fresh biomass per plant (g). (K) Edible fresh biomass per plant (g). (L) Harvest index. (M) Dry matter content (%). (N) Days to 50% flowering (days). The mean of each population of regions of origin is indicated by a dotted line.

The distribution frequency of all agronomic traits according to the regions of origin of the lines is presented in Fig 1. Significant differences (p < 0.05) were observed among the regions of origin for all fourteen investigated traits (Fig 1, S2 Table). East African genotypes followed by the Southern African genotypes outperformed West African and Asian genotypes in stem diameter, number of primary branches, petiole length, total fresh biomass and edible fresh biomass, and days to 50% flowering. The Southern African genotypes had longer central leaflet and broader leaf. In contrast, the West African genotypes had longer primary branch and higher dry matter content, whereas the Asian genotypes had broader central leaflet and a higher harvest index (Fig 1).

### Variance components, heritability and genetic gain estimates of biomass and related traits

Significant genotypic variances ($\sigma_{G}^{2}$) were observed for all traits (across years, Table 4 and each year, S3 Table), while genotype × year interaction variances ($\sigma_{G\times Y}^{2}$) were significant for stem diameter, primary branch length, number of primary branches, leaf width and area, petiole length, harvest index and days to 50% flowering (Table 4). For all traits, genotype × year interaction variances were lower than genotypic variances ($\sigma_{G}^{2}$). The broad-sense heritability was high for all traits and ranged between 0.64 ± 0.09 (edible biomass per plant) and 0.87 ± 0.03 (petiole length) (Table 4). Genetic gains at 5% selection intensity were variable (Table 4, S3 Table). Estimates of genetic gains over the mean of the current population were low for dry matter content (13.75%) and high for primary branch length (117.36%). Specifically, significant genetic gains (> 50%) were observed for the number of primary branches, leaf area, and total and edible fresh biomass. Variable genotypic and phenotypic coefficients of variation were observed for all fourteen traits. Dry matter content had low phenotypic and genotypic coefficients of variation (< 10%), while days to 50% flowering, central leaflet width and harvest index had medium phenotypic and genotypic coefficients of variation (ranging between 10 and 20%). Other traits displayed high phenotypic and genotypic coefficients of variation. In comparison, trends in error coefficients of variation for all traits were similar to those of phenotypic and genotypic coefficients of variation (Table 4).

**Table 4:** Estimates of genetic parameters for biomass and related traits in 71 advanced lines of *Gynandropsis gynandra* evaluated over two years.

Traits	$\sigma_{G}^{2}$	$\sigma_{G\times Y}^{2}$	$\sigma_{e}^{2}$	*H* ^2^	$\sigma_{P}^{2}$	GA	GAM	GCV	PCV	ECV
**StDiam**	3.95 ± 0.95 ***	1.03 ± 0.56 *	3.14 ± 0.58	0.75 ± 0.06	5.25 ± 0.91	3.55	35.85	20.07	23.14	17.90
**PHeight**	204.79 ± 48.23 ***	32.13 ± 29.50	188.05 ± 36.63	0.76 ± 0.06	267.86 ± 46.02	25.78	36.68	20.36	23.29	19.51
**PBrLeng**	394.96 ± 86.71 ***	70.72 ± 37.40 *	223.93 ± 40.54	0.81 ± 0.05	486.30 ± 84.26	36.89	117.36	63.22	70.15	47.60
**NPBr**	11.79 ± 2.38 ***	1.37 ± 0.80 *	4.91 ± 0.91	0.86 ± 0.04	13.70 ± 2.35	6.56	62.06	32.48	35.01	20.96
**CtLleng**	1.85 ± 0.39 ***	0.19 ± 0.17	1.10 ± 0.21	0.83 ± 0.04	2.22 ± 0.38	2.56	35.86	19.07	20.88	14.68
**CtLwid**	0.27 ± 0.06 ***	0.03 ± 0.03	0.21 ± 0.04	0.80 ± 0.05	0.33 ± 0.06	0.96	30.26	16.40	18.31	14.50
**Lwid**	5.59 ± 1.25 ***	1.50 ± 0.60 **	2.83 ± 0.53	0.79 ± 0.05	7.04 ± 1.21	4.34	39.79	21.69	24.35	15.44
**Ptillen**	6.52 ± 1.31 ***	0.84 ± 0.40 **	2.34 ± 0.44	0.87 ± 0.03	7.52 ± 1.29	4.90	45.04	23.48	25.23	14.07
**LfArea**	424.98 ± 92.24 ***	106.21 ± 41.98 **	186.06 ± 35.78	0.81 ± 0.05	524.60 ± 89.59	38.22	71.90	38.78	43.08	25.66
**FBiom**	732.44 ± 188.27 ***	180.28 ± 138.26	895.93 ± 171.22	0.70 ± 0.08	1046.56 ± 175.74	46.64	69.13	40.11	47.95	44.37
**EDBiom**	97.52 ± 27.31 ***	25.87 ± 22.96	170.46 ± 30.26	0.64 ± 0.09	153.07 ± 24.92	16.24	57.58	35.02	43.87	46.30
**HI**	0.006 ± 0.002 ***	0.002 ± 0.001 **	0.005 ± 0.001	0.70 ± 0.08	0.01 ± 0.00	0.13	27.91	16.19	19.36	15.18
**DM**	0.76 ± 0.21 ***	0.20 ± 0.16	1.10 ± 0.20	0.67 ± 0.09	1.13 ± 0.20	1.46	13.75	8.17	10.00	9.85
**DFlow**	50.68 ± 13.03 ***	28.63 ± 7.57 ***	20.29 ± 4.32	0.72 ± 0.07	70.07 ± 12.34	12.47	20.75	11.85	13.93	7.50

StDiam: Stem diameter (mm), PHeight: Plant height (cm), PBrLeng: Primary branch length (cm), NPBr: Number of primary branches, CtLleng: Central leaflet length (cm), CtLwid: Central leaflet width (cm), Lwid: Leaf width (cm), Ptillen: Petiole length (cm), LfArea: Leaf area (cm^2^), FBiom: Total fresh biomass per plant (g), EDBiom: Edible fresh biomass per plant (g), HI: Harvest index, DM: Dry matter content (%), DFlow: Days to 50% flowering (days), $\sigma_{e}^{2}$ = residual variance, $\sigma_{G}^{2}$ = genotypic variance, $\sigma_{G\times Y}^{2}$ = genotype × year variance, $\sigma_{P}^{2}$ = phenotypic variance, *H*^2^ = broad-sense heritability, GA: Genetic advance; GAM: Genetic advance over mean, GCV: Coefficient of genotypic variation; PCV: Coefficient of phenotypic variation, ECV: Residual coefficient of variation.

***, **, *: Significantly different from zero at the 0.001, 0.01, and 0.05 probability level, respectively. *ns*: Not significantly different from zero at the 0.05 level of probability.

### Association among plant biomass and related traits

**Fig 2:**
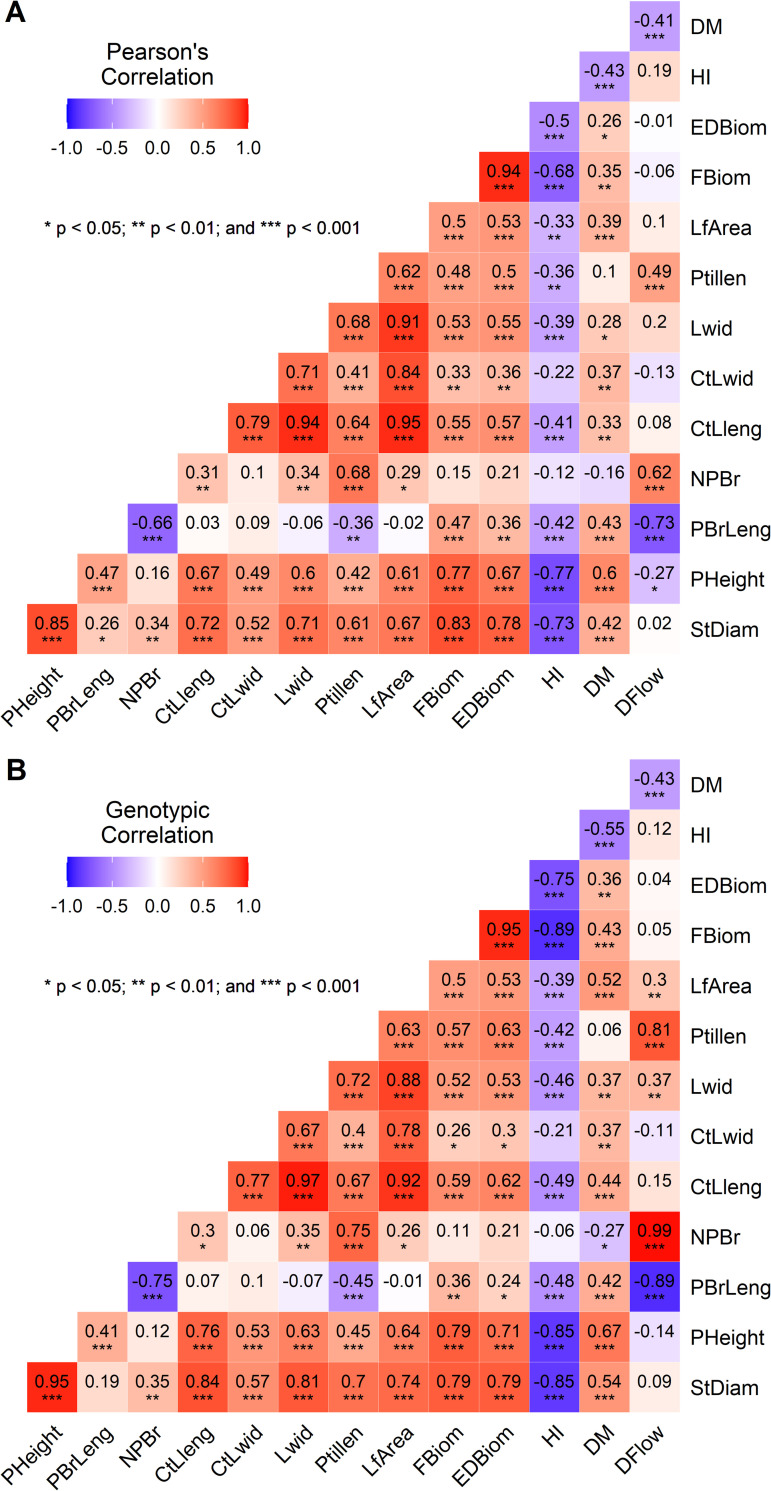
Plots of Pearson’s phenotypic (A) and genotypic (B) correlation coefficients for fourteen agronomic traits of 71 advanced lines of *Gynandropsis gynandra*. StDiam: Stem diameter (mm), PHeight: Plant height (cm), PBrLeng: Primary branch length (cm), NPBr: Number of primary branches, CtLleng: Central leaflet length (cm), CtLwid: Central leaflet width (cm), Lwid: Leaf width (cm), Ptillen: Petiole length (cm), LfArea: Leaf area (cm^2^), FBiom: Total fresh biomass per plant (g), EDBiom: Edible fresh biomass per plant (g), HI: Harvest index, DM: Dry matter content (%), DFlow: Days to 50% flowering (days).

Significant phenotypic and genotypic correlation coefficients were observed among the fourteen agronomic traits (Fig 2). While the phenotypic correlation coefficients ranged from -0.77 to 0.95, the genotypic correlation coefficients varied between -0.89 and 0.99. Similar trends were observed for the two types of correlation. For instance, a highly significant and positive correlation was observed between edible and total fresh biomass per plant at both phenotypic (r = 0.94, p < 0.001) and genotypic (r = 0.95, p < 0.001) levels (Fig 2). Total and edible biomass per plant had strong and positive correlations with plant height and stem diameter and positive and moderate correlations with all leaf-related traits (central leaflet length, central leaflet width, leaf width, petiole length and leaf area) and primary branch length. There were moderate to strong positive correlations among leaf traits, with leaf area being strongly and positively correlated with central leaflet length, central leaflet width and leaf width. Days to 50% flowering had moderate and positive correlations with the number of primary branches and petiole length but had a strong and negative correlation with the primary branch length and a moderate and negative correlation with dry matter content (Fig 2). The harvest index had negative and significant correlations with most traits, with strong correlations with stem diameter, plant height, and total fresh biomass. Additionally, the harvest index had moderate and negative correlations with edible plant biomass, dry matter content, primary branch length and leaf traits (central leaflet length, leaf width, and leaf area). Dry matter content had moderate and positive correlations with plant height, stem diameter, number of primary branches, total and edible fresh biomass per plant and leaf traits. The number of primary branches had a strong and negative correlation with primary branch length. A strong and positive correlation was observed between stem diameter and plant height. In addition, stem diameter and plant height had a moderate to strong positive correlation with leaf traits (Fig 2).

### Multivariate analysis of biomass and related traits in spider plant

**Fig 3:**
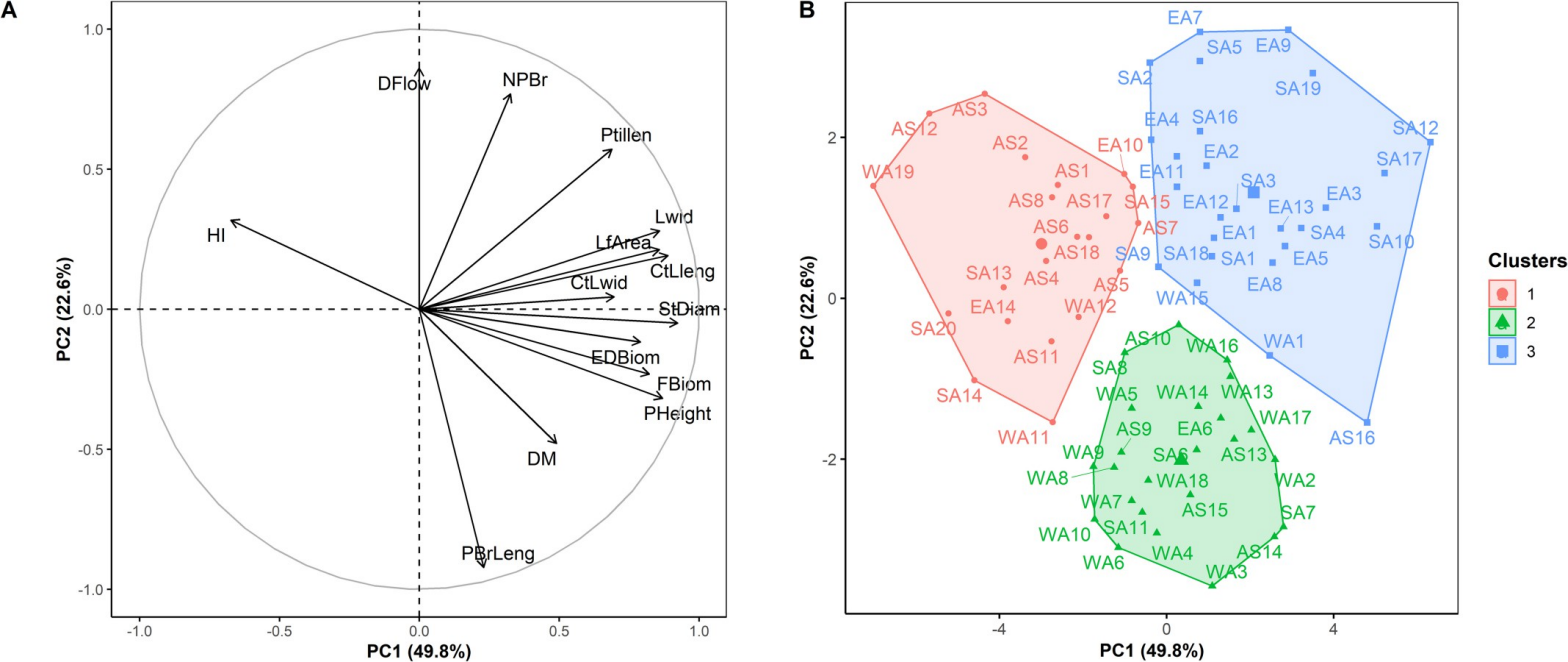
Correlation circle (A) and factor map (B) showing the clustering pattern of 71 advanced lines of *Gynandropsis gynandra* based on the hierarchical clustering on principal components analysis (HCPC). Cluster 1 (n = 21), Cluster 2 (n = 24) and Cluster 3 (n = 26). StDiam: Stem diameter (mm), PHeight: Plant height (cm), PBrLeng: Primary branch length (cm), NPBr: Number of primary branches, CtLleng: Central leaflet length (cm), CtLwid: Central leaflet width (cm), Lwid: Leaf width (cm), Ptillen: Petiole length (cm), LfArea: Leaf area (cm^2^), FBiom: Total fresh biomass per plant (g), EDBiom: Edible fresh biomass per plant (g), HI: Harvest index, DM: Dry matter content (%), DFlow: Days to 50% flowering (days). AS: Asia; EA: East Africa; SA: Southern Africa; WA: West Africa.

To assess the relationship among genotypes, we first performed a principal component analysis. The results of the principal component analysis revealed that the first two components explained 72.43% of the total variation in the biomass and related traits and correlated with most traits (Fig 3A). Traits significantly associated with the first principal component (explaining 49.79% of the total variation) included stem diameter, plant height, leaf traits (central leaflet length, central leaflet width, leaf width, petiole length and leaf area), biomass (total and edible fresh biomass par plant) and harvest index. Principal component 1 was negatively correlated with harvest index but positively correlated with all other traits. Principal component 2 was positively and significantly associated with days to 50% flowering and the number of branches but negatively correlated with the primary branch length (Fig 3A).

**Fig 4:**
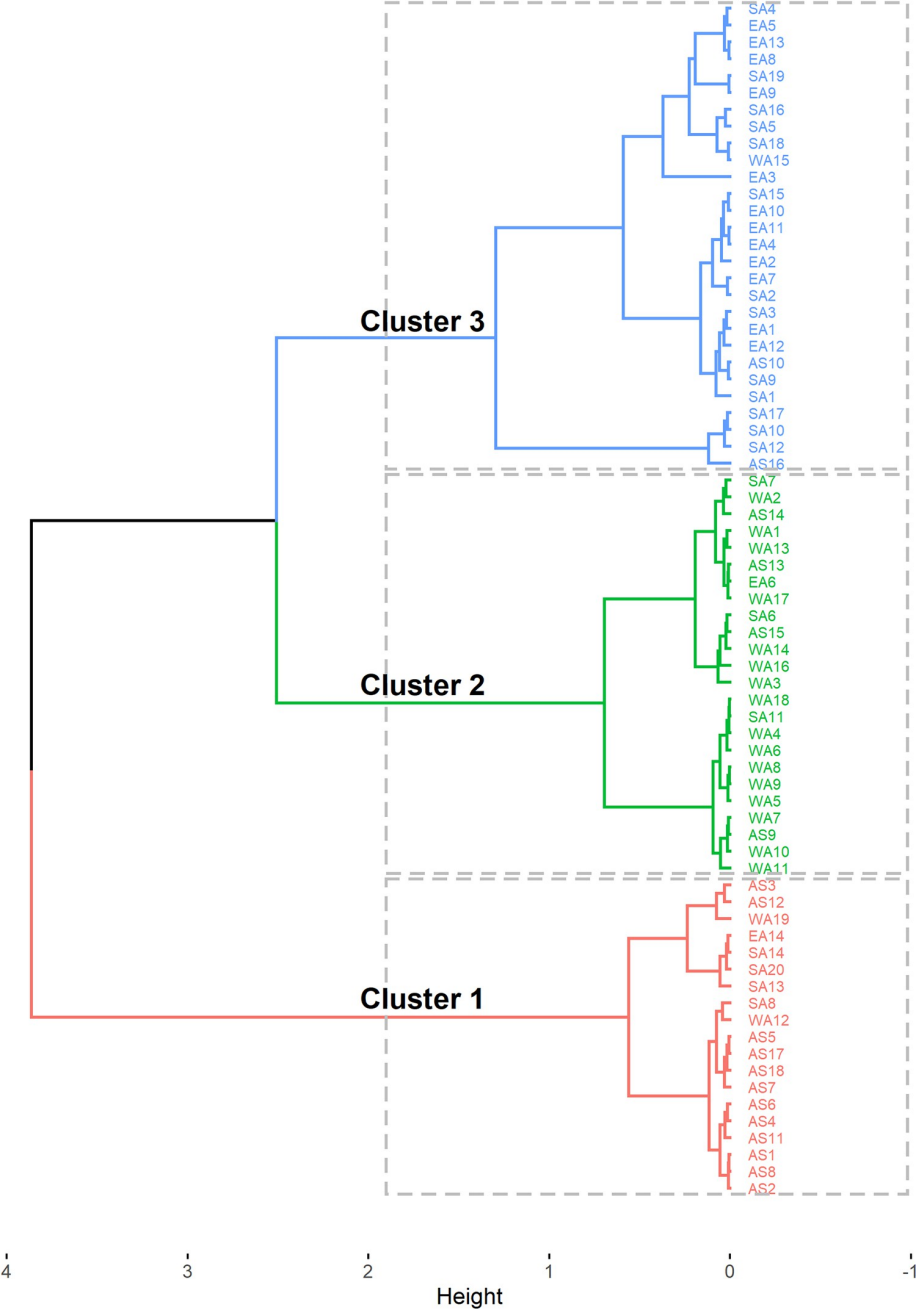
Dendrogram showing the lines constituting the three clusters. AS: Asia; EA: East Africa; SA: Southern Africa; WA: West Africa.

Clustering pattern analysis using hierarchical clustering on principal components classified the lines into three clusters (Figs 3B and 4). A significant difference was observed among the clusters for all traits (Table 5). Cluster 1 (29.58% of all lines) encompassed mainly Asian lines (66% of all Asian lines) with some from other regions and was characterized by less vigorous plants, with a moderate number of short primary branches, low biomass productivity and dry matter content, relatively late flowering time, small leaves, and high harvest index (Table 5). Cluster 2 included mainly lines originating from West Africa (73.68% of all West African lines) and some from other regions. Genotypes in cluster 2 had high dry matter content, long primary branches, high biomass productivity, low number of primary branches, moderate vigor, medium leaf size and flowered early. Cluster 3, mainly composed of lines from East and Southern Africa (88.46% of all lines in the cluster), was characterized by late flowering and vigorous plants, a high number of short primary branches, high biomass productivity, broad leaves, moderate dry matter content and a low harvest index (Table 5).

**Table 5:** Phenotypic descriptors of *Gynandropsis gynandra*’s clusters.

Phenotypic descriptors	Cluster 1 (n = 21)	Cluster 2 (n = 24)	Cluster 3 (n = 26)	F Value	All the germplasm
	Asia n = 12	Asia n = 5	Asia n = 1		Asia n = 18
	East Africa n = 2	East Africa n = 1	East Africa n = 11		East Africa n = 14
	Southern Africa n = 4	Southern Africa n = 4	Southern Africa n = 12		Southern Africa n = 20
	West Africa n = 3	West Africa n = 14	West Africa n = 2		West Africa n = 19
**StDiam: stem diameter (mm)**	*7*.*84 ± 0*.*21* c	10.29 ± 0.20 b	**11.21 ± 0.21 a**	69.53 ***	9.9 ± 0.2
**PHeight: plant height (cm)**	*56*.*64 ± 2*.*07* b	**75.67 ± 1.51 a**	**76.31 ± 1.86 a**	35.52 ***	70.27 ± 1.47
**NPBr: number of primary branches**	10.02 ± 0.42 b	*7*.*97 ± 0*.*38* c	**13.42 ± 0.51 a**	40.12 ***	10.57 ± 0.37
**PBrLeng: primary branch length (cm)**	*19*.*21 ± 2*.*13* b	**52.12 ± 1.88 a**	*22*.*22 ± 2*.*02* b	81.74 ***	31.44 ± 2.09
**CtLleng: central leaflet length (cm)**	*6*.*18 ± 0*.*18* c	6.99 ± 0.16 b	**8.06 ± 0.24 a**	21.90 ***	7.14 ± 0.15
**CtLwid: central leaflet width (cm)**	*2*.*94 ± 0*.*10* b	3.19 ± 0.06 ab	**3.30 ± 0.10 a**	4.14 *	3.16 ± 0.05
**Lwid: leaf width (cm)**	*9*.*23 ± 0*.*27* c	10.41 ± 0.28 b	**12.69 ± 0.36 a**	31.68 ***	10.9 ± 0.25
**Ptillen: petiole length (cm)**	*9*.*57 ± 0*.*36* b	*9*.*53 ± 0*.*31* b	**13.16 ± 0.32 a**	42.14 ***	10.87 ± 0.28
**LfArea: leaf area (cm** ^**2**^ **)**	*40*.*00 ± 2*.*32* c	49.72 ± 2.06 b	**66.97 ± 4.00 a**	20.13 ***	53.16 ± 2.18
**FBiom: total fresh biomass per plant (g)**	*40*.*01 ± 2*.*08* b	**77.67 ± 2.63 a**	**80.23 ± 3.57 a**	54.99 ***	67.47 ± 2.69
**EDBiom: edible fresh biomass per plant (g)**	*19*.*42 ± 0*.*84* b	**30.96 ± 0.98 a**	**32.75 ± 1.48 a**	35.38 ***	28.2 ± 0.95
**HI: harvest index**	**0.54 ± 0.01 a**	*0*.*44 ± 0*.*01* b	*0*.*44 ± 0*.*01* b	37.52 ***	0.47 ± 0.01
**DM: dry matter content (%)**	*10*.*27 ± 0*.*10* b	**10.92 ± 0.13 a**	10.69 ± 0.16 ab	5.61 **	10.64 ± 0.08
**DFlow: days to 50% flowering (days)**	61.00 ± 1.05 a	*54*.*73 ± 0*.*52* b	**64.33 ± 1.03 a**	30.72 ***	60.1 ± 0.7

Values in bold and italics indicate clusters’ means that are significantly greater and lower than the overall means for all accessions, respectively, and describe the given cluster. Values within a row followed by the different letters are significantly different according to Tukey’s HSD at P ≤ 0.05.

***, **, * indicate significance at the 0.001, 0.01, and 0.05 probability level, respectively.

## Discussion

Genetic variation is the foundation of any plant breeding program. Significant and origin-driven variation has been reported in *Gynandropsis gynandra* for plant morphology [3,55], secondary metabolite concentrations [9], seed germination, mineral composition and morphology [39], leaf vitamin contents [3], antioxidant activity [12], and photosynthesis traits [56]. Morphological traits with significant variation were related to plant architecture (plant height, number of primary branches, plant habit, stem hairiness and colour), leaf size (leaf area, leaflet length and width, petiole length, leaflet shape), leaf colour, days to 50% flowering, germination (percentage and mean time), pod characteristics (pod length and width, number of seeds per pod), seed size (length, width, perimeter, area), 1000-seed weight, flower traits (androphore length, filament length, pedicel length, gynophore length), and biomass (total shoot fresh and dry weight, leaf fresh and dry weight) [3,4,39,55]. In addition, phenotypic differentiation among diverse accessions of *G*. *gynandra* was found to be associated with the genetic makeup of the genotypes [57,58]. While Omondi et al. [58] differentiated advanced lines and genebank’s accessions from farmer cultivars using simple sequence repeats (SSR) markers, Sogbohossou [57] observed genomic differentiation among accessions from West Africa, East/Southern Africa and Asia. Our study revealed that four generations of selfing maintained significant variation and membership in their group of origin, strengthening the hypothesis of geographical signature in cleome genetic diversity. We observed highly significant variation among advanced lines for biomass productivity, growth traits, leaf traits and flowering time in *Gynandropsis gynandra*. Similar observations for morphological traits have also been reported for worldwide accessions [3,55], East and Southern African accessions and cultivars [4], and accessions from South Africa and Kenya [54], Ghana [50], and Burkina-Faso [51]. This significant variation represents a valuable resource for sustainable and successful breeding programs for the species.

On the other hand, the average and the highest total fresh biomass in the present study were higher than those reported by Omondi et al. [4] in East-Southern African genotypes but slightly lower than those of Kiebre et al. [51] for accessions from Burkina-Faso. The difference might be attributable to the genotypes, agricultural practices, and environment since those authors evaluated their germplasm in the field. For instance, agronomic practices such as planting density, type and fertilizer application rates, planting date, stage of transplanting, harvesting frequency and techniques (cutting, uprooting whole plants, defoliation) significantly affect growth and biomass yield in *G*. *gynandra* [43–49]. Therefore, genotype performance should be investigated under different agricultural practices considering farmers’ practices in target environments.

The clustering analysis identified three groups, each dominated by lines derived from accessions originating from different geographical regions. Clusters 1, 2 and 3 were dominated by lines derived from Asian, West African, and East/Southern African accessions, respectively. The clustering results were supported by the significant differences observed among regions of origin for all fourteen investigated traits. These results align with previous reports on the association between the geographical origin and the morphology of the accessions of *G*. *gynandra* [3,55]. Specifically, Sogbohossou et al. [3] identified three distinct groups similar to those of this study: East-Southern African accessions (tall plants with broad leaves), Asian accessions (short plants with broad leaves) and West African accessions (short plants with small leaves). Furthermore, the genetic constitution could be the main driver of this clustering, as Sogbohossou [57] reported genomic differentiation between Asian, West African and East/Southern African accessions. This clustering pattern might reflect the local adaptation of the species in response to environmental/climatic factors and different uses by local communities.

Farmers’ preferred traits in *G*. *gynandra* include high leaf yield and related traits (plant height and the number of leaves), broad leaves and late flowering [32,59–61]. We observed that East and Southern African lines combined several farmers’ preferred traits such as broad leaves, late flowering and high biomass, while West African genotypes had high biomass and dry matter content. Based on biomass productivity, East, Southern and West African genotypes were similar and outperformed the Asian accessions, which could be in response to ancient domestication or advanced selection for biomass occurring in these regions compared with Asia. Intensive utilization of the species as a leafy vegetable has been reported in Africa rather than Asia. In several Asian countries, the species was mainly reported as weeds and rarely cultivated [84,85] and primarily used in traditional medicine [19,22]. In contrast, although the species still grows as weeds, it is cultivated in many African countries for its leaves as vegetables [29]. In Africa, the semi-cultivated status of *G*. *gynandra* was reported earlier in the 1950s [86]. The domestication of the species might have first started in Eastern and Southern Africa, as its weed status was quickly converted to cultivated species [87]. West African genotypes had similar biomass yields as the East and Southern African genotypes, suggesting West Africa as a secondary domestication hotspot for the species, while domestication and selection are still at the earlier stage or might not have started in Asia. Feodorova et al. [26] support these findings, by suggesting that the speciation event of *G*. *gynandra* might have occurred in South Africa. Using genome sequencing, Sogbohossou [57] suggested the African origin of the species, with Asian and West African populations being closed and recently divergent from East and Southern African populations. More investigations are needed to clarify the origin of the species as well as its route of colonization.

Heritability is important in breeding, as it helps in predicting the efficiency of the selection. Broad-sense heritability (H^2^) measures the proportion of the total phenotypic variation attributable to the variance of genetic values [88]. High broad-sense heritability estimates (> 0.60) were observed for all investigated traits, showing that phenotypic variation observed among genotypes is mostly due to genotypic variation. More importantly, we also observed low genotype × year interaction variance compared with genotypic variance. We therefore hypothesize that phenotypes can accurately predict genotypes, but this should be confirmed with multi-environmental trials. Similarly, high broad-sense heritability estimates were reported for stem diameter, plant height, number of primary branches, leaf biomass, leaf area, leaflet length and width, and days to 50% flowering in the species [54,89]. This suggests that high genetic advancement is achievable for biomass and related traits in the species. As a consequence, we observed significant expected genetic gain at a selection intensity of 5%, showing that significant improvement would be possible through direct phenotypic selection, particularly for total fresh biomass, edible fresh biomass, the number of primary branches and leaf area. These findings concur with earlier reports in *G*. *gynandra* for biomass yield and related traits [89]. The low genetic gain observed for dry matter content might suggest that selecting this trait might be difficult, as low variability was also observed. More genetic material is needed to broaden the available variability.

Genotype × year interaction variances were significant for stem diameter, primary branch length, number of primary branches, leaf width and area, petiole length, harvest index and days to 50% flowering. This is showing that these traits were influenced not only by the genotype but also by the interaction between genotype and year. As agronomic practices were the same during the two years, the differential environmental conditions between 2020 and 2021 could play a significant role in the significance of genotype × year interaction. Potential environmental factors that might influence these traits could include the temperature, the relative humidity, the light intensity and the day length (photoperiod). Imbamba and Tieszen [90] found that the photosynthesis rate in spider plant increase with light intensity (from 200 to 2000 μmol m^-2^ s^-1^) and that 2000 μmol m^-2^ s^-1^, which is close to full sunlight, does not saturate photosynthesis in *G*. *gynandra* as it is a C4 plant. Similarly, Kocacinar [91] observed an increase in the net photosynthetic rate and stomatal conductance with increasing light intensity. Specifically, the genotype × year interaction variance was highly significant for flowering time compared to other traits. This is attributable to the day length sensitivity of the species. Zorde et al. [92] observed significant variation in days to flowering between the greenhouse (10–182 days) and field (20–57 days) trials in Arusha due to the differential day length and light intensity. In fact, the plants were grown under daylight conditions between 11:52–12:17 hours of daylight (field) compared to 14 hours in the greenhouse. The authors pointed out that light intensity may have further explained this as differences in light intensity significantly affect flowering time and yield [93] and the field evaluation might receive more intense light. The leaf temperature also significantly influences the rates of CO_2_ assimilation, and the species requires high temperature (30–40°C) to attain maximum photosynthesis, playing a key role in the species’ growth and biomass productivity. On the other hand, the year significantly affected some of these traits, implying that these traits might vary with year. In addition, the significant genotype × year interaction indicated that the genotypes’ performance was not consistent across environments, and selection should consider the interaction effect when selecting genotypes. However, evaluation in additional environments, particularly in field conditions, is required to better decipher the genotype by environment interaction in the species.

Understanding the association between traits offers an opportunity for efficient and simultaneous selection. Both phenotypic and genotypic correlations showed similar trends. In the present study, the correlation between total fresh biomass and edible fresh biomass was strong, positive and significant. In addition, these two traits were highly and positively correlated with plant height and stem diameter, suggesting that selection for vigorous and tall plants will lead to high-yielding cultivars. This might be accompanied by broad leaves resulting from the positive and moderate association between biomass and leaf-related traits (central leaflet length, central leaflet width, leaf width, petiole length, leaf area). Previous findings corroborated these results as a positive and strong correlation of leaf biomass with plant height, stem diameter, leaf length and width and petiole length [89]. Similarly, Kangai Munene et al. [54] and Mosenda et al. [53] observed a positive and strong association between the number of leaves per plant and plant height [54]. Such a positive association between these traits imply that simultaneous and direct selection for such farmers’ desired traits would be possible. This association could result from pleiotropic or linked genes controlling biomass, plant height, stem diameter, and leaf traits in the species. Using an F2 population, Sogbohossou [57] found a single QTL for plant height and two for leaf area, and this plant height QTL and one QTL for leaf area were colocalized on the same linkage group, with potential pleiotropic effects of a candidate gene, although the author recommended the validation of the QTLs.

The number of primary branches was positively correlated with days to 50% flowering, suggesting that late flowering plants had more branches. In contrast, primary branch length had a negative and significant correlation with days to 50% flowering and number of branches, showing the existence of a trade-off between the number of primary branches, the primary branch length and days to 50% flowering in the species. After flowering, plants allocate resources for lateral branch growth, therefore, the plant can achieve higher biomass either by flowering early and developing long branches or delaying flowering to produce more branches. This might explain why West African genotypes had similar biomass yields to East/Southern genotypes, which are late flowering with a high number of short branches. This calls for an in-depth investigation to understand resource allocation in the species and genes involved in flowering time, branch development, and plant architecture. To this end, developing mapping populations using genotypes from all clusters will be insightful.

In this study, the harvest index was negatively associated with plant biomass and most other agronomic traits, suggesting that selection for the harvest index might be difficult. However, using appropriate agronomic traits, such as early harvesting, could help improve the harvest index. Frequent harvesting (e.g., every week or two weeks) might increase biomass productivity and extend the harvesting period. This would strongly depend on the regrowth ability of the genotype. An evaluation of the germplasm under different agronomic practices, including harvesting techniques and frequency, is required, as suggested by Houdegbe et al. [43]. Assessing the regrowth ability would be crucial, particularly in West Africa, where cutting is the frequent harvesting technique employed by farmers and genotypes with several cuttings are desired [94]. In this case, the ability to predict yield for the subsequent harvest should be investigated through genetic correlation analysis.

Dry matter content is associated with shelf life and determines the vegetable’s post-harvest behaviour [95–97]. The moderate and significant association of dry matter content with plant biomass, growth traits and leaf traits suggested that increasing the leaf area might not affect dry matter content in the species. In contrast, the negative association between days to 50% flowering and dry matter content showed that late flowering plants might have low dry matter content with reduced shelf life, suggesting plausible linkage drag between flowering time and dry matter accumulation in the species. Similarly, a negative correlation was observed between dry matter content and days to silking in maize for biogas production [98]. Such an association could be investigated using mapping populations developed between West and East/Southern African genotypes. In addition, broadening the narrow genetic variation for dry matter content is needed through extensive germplasm collections, introductions and characterization.

Overall, considering farmers’ preferred traits, genotypes in cluster 3 and somewhat cluster 2 are good candidates for cultivar release and breeding programs. Superior genotypes from these clusters with multiple improved traits included SA10, SA12, SA17 (edible biomass, stem diameter, number of primary branches, plant height, and leaf area and dry matter content), EA12, EA3 (fresh and edible biomasses and days to flowering), AS14, AS16, WA17, WA3 (fresh biomass, dry matter content and primary branch length), EA7, SA2, and EA9 (days to 50% flowering time and number of primary branches). An intensive field evaluation of these genotypes through multi-environment trials within each region would help in understanding the genotype-by-environment interaction in the species and whether to breed for specific or broad adaptation. Furthermore, establishing the link between the phenotype and genotype is required to help implement marker-assisted selection in the species. Genome-wide association studies (GWAS) can be implemented to decipher genes associated with functional and farmers’ preferred traits and would serve in the validation of QTLs reported by Sogbohossou [57] on flowering time, plant height, and leaf area. The best genotypes from each cluster could be involved in studies to estimate the narrow-sense heritability and determine gene action controlling the key traits using factorial mating designs such as diallel and North Carolina mating designs. In addition, assessing the potential hybrid vigour in the species would help design efficient breeding strategies for ideal cultivar development. Association of these traits with nutritional traits is needed. Evaluation of these genotypes under different disease and pest pressures and biotic stresses is required, particularly in the current changing climate.

## Conclusion

The present study revealed that the biomass potential of advanced lines of spider plant was associated with their geographical origin, thus strengthening the hypothesis of geographical signature in cleome diversity. West and East/Southern African lines had higher biomass productivity than Asian lines, suggesting advanced selection and domestication in Africa than Asia for biomass. The significant genetic variation, high broad-sense heritability, genetic gain and positive correlation between plant biomass and related traits provides the opportunity for positive and simultaneous selection, especially for farmers’ preferred traits such as biomass yield, leaf size, flowering time and the number of branches. The genotypes SA10, SA12, SA17, EA12, EA3, AS14, AS16, WA17, WA3, EA7, SA2, EA9 are superior for multiple farmers’ desired traits and good candidates for breeding programs and cultivars release. Further studies should target multi-environment trials to determine genotype by environment interaction effect, determine the genotypes’ response to different agronomic practices such as cutting, fertilization considering the locally available resources, identify gene action and genes controlling farmers preferred traits and evaluate the germplasm tolerance to biotic and abiotic stress. Additionally, the association of plant biomass and related traits with key nutritional traits such as minerals is required to ensure the quality of the end products for users.

## Supporting information

S1 Table Phenotypic values of fourteen agronomic traits among regions of origin of 71 advanced lines of *Gynandropsis gynandra* evaluated across two years. (CSV)

S2 Table Variation in phenotypic values of fourteen agronomic traits among regions of origin of 71 advanced lines of *Gynandropsis gynandra*. (DOCX)

S3 Table Estimates of genetic parameters for biomass and related traits in 71 advanced lines of *Gynandropsis gynandra* evaluated in 2020 and 2021. (DOCX)
