# Supplementary material for: Phenotypic variation in biomass and related traits among four generations advanced lines of Cleome (Gynandropsis gynandra L. (Briq.))
Source: PLoS One. 2022 Oct 12;17(10):e0275829. doi: 10.1371/journal.pone.0275829 (PMC9555646; doi:10.1371/journal.pone.0275829)
Supplement: S2 Table — (DOCX) [file pone.0275829.s002.docx]

**S2 Table. Variation in phenotypic values of fourteen agronomic traits among regions of origin of 71 advanced lines of *Gynandropsis gynandra***

| **Phenotypic traits** | **Regions of origin** | | | | **ANOVA or Kruskal–Wallis test (P values)** |
| --- | --- | --- | --- | --- | --- |
|  | **Asia** | **East Africa** | **Southern Africa** | **West Africa** |  |
| **StDiam: stem diameter (mm)** | 8.58±0.28b | **10.95±0.36a** | **10.43±0.35a** | **10.08±0.3a** | p < 0.001 |
| **PHeight: plant height (cm)** | 63.43±2.43b | **70.97±2.64ab** | **73.35±2.63a** | **74.44±2.05a** | p = 0.006 |
| **NPBr: number of primary branches** | 10.16±0.3b | **13.74±0.54a** | 11.92±0.57ab | 7.99±0.27c | p < 0.001 |
| **PBrLeng: primary branch length (cm)** | 26.3±2.82b | 19.99±2.61b | 24.14±2.85b | **49.92±2.4a** | p < 0.001 |
| **CtLleng: central leaflet length (cm)** | 6.68±0.17b | 7.45±0.21ab | **7.78±0.23a** | 6.8±0.19b | p = 0.001 |
| **CtLwid: central leaflet width (cm)** | 3.34±0.07a | 3.02±0.08b | 3.17±0.1ab | 3.08±0.07ab | p = 0.027 |
| **Lwid: leaf width (cm)** | 9.72±0.26b | **11.79±0.39a** | **12.2±0.41a** | 10.1±0.32b | p < 0.001 |
| **Ptillen: petiole length (cm)** | 10.21±0.23b | **13.4±0.38a** | **12.22±0.36a** | 8.74±0.26c | p < 0.001 |
| **LfArea: leaf area (cm^2^)** | 46.91±2.3b | 55.86±3.39ab | **63.64±3.91a** | 46.89±2.2b | p = 0.014 |
| **FBiom: total fresh biomass per plant (g)** | 46.52±4.16b | **85.22±7.07a** | **72.28±4.91a** | **70.1±3.72a** | p < 0.001 |
| **EDBiom: edible fresh biomass per plant (g)** | 21.1±1.61b | **34.27±3.06a** | **31.18±2.02a** | **28.5±1.47a** | p < 0.001 |
| **HI: harvest index** | **0.52±0.02a** | 0.41±0.01c | 0.47±0.01b | 0.45±0.01bc | p < 0.001 |
| **DM: dry matter content (%)** | 10.57±0.19b | 10.33±0.15b | 10.62±0.18ab | **11.05±0.16a** | p = 0.016 |
| **DFlow: days to 50% flowering (days)** | 56.5±1.71bc | **69.25±1.69a** | 62.54±1.6b | 55.99±1.19c | p < 0.001 |

Values within a row followed by the different letters are significantly different according to Tukey’s HSD or Dunn’s test at p < 0.05.
