# Supplementary material for: Phenotypic variation in biomass and related traits among four generations advanced lines of Cleome (Gynandropsis gynandra L. (Briq.))
Source: PLoS One. 2022 Oct 12;17(10):e0275829. doi: 10.1371/journal.pone.0275829 (PMC9555646; doi:10.1371/journal.pone.0275829)
Supplement: S3 Table — (DOCX) [file pone.0275829.s003.docx]

**S3 Table. Estimates of genetic parameters for biomass and related traits in 71 advanced lines of *Gynandropsis gynandra* evaluated in 2020 and 2021.**

| **Traits** | **Year** | $\boldsymbol{\sigma}_{\boldsymbol{G}}^{\boldsymbol{2}}$ | $\boldsymbol{\sigma}_{\boldsymbol{e}}^{\boldsymbol{2}}$ | $\boldsymbol{\sigma}_{\boldsymbol{P}}^{\boldsymbol{2}}$ | $\boldsymbol{H}^{\boldsymbol{2}}$ | **Mean** | **GA** | **GAM** | **GCV** | **PCV** | **ECV** |
| --- | --- | --- | --- | --- | --- | --- | --- | --- | --- | --- | --- |
| **StDiam** | 2020 | 5.21±1.11*** | 2.36±0.41 | 6.39±1.32 | 0.81±0.10 | 10.4±0.99 | 4.24 | 40.79 | 21.93 | 24.3 | 14.78 |
|  | 2021 | 5.06±1.35*** | 3.67±0.73 | 6.90±1.71 | 0.73±0.20 | 9.41±1.23 | 3.97 | 42.22 | 23.93 | 27.93 | 20.38 |
| **PHeight** | 2020 | 229.67±50.92*** | 112.68±22.03 | 286.01±61.93 | 0.80±3.52 | 73.77±8.49 | 27.98 | 37.92 | 20.54 | 22.92 | 14.39 |
|  | 2021 | 302.38±80.46*** | 208.81±42.59 | 406.78±101.75 | 0.74±8.18 | 66.20±9.43 | 30.88 | 46.65 | 26.27 | 30.47 | 21.83 |
| **PBrLeng** | 2020 | 517.60±102.16*** | 148.16±28.82 | 591.68±116.56 | 0.87±3.18 | 31.17±8.57 | 43.83 | 140.62 | 72.98 | 78.03 | 39.05 |
|  | 2021 | 337.28±96.55*** | 295.71±58.98 | 485.13±126.04 | 0.70±12.56 | 30.50±10.77 | 31.54 | 103.44 | 60.22 | 72.23 | 56.39 |
| **NPBr** | 2020 | 12.47±2.44*** | 3.54±0.67 | 14.24±2.78 | 0.88±0.10 | 10.69±1.28 | 6.81 | 63.67 | 33.03 | 35.30 | 17.6 |
|  | 2021 | 13.36±3.07*** | 5.42±1.22 | 16.07±3.68 | 0.83±0.21 | 10.63±1.70 | 6.87 | 64.56 | 34.37 | 37.70 | 21.89 |
| **CtLleng** | 2020 | 2.06±0.41*** | 0.68±0.13 | 2.40±0.48 | 0.86±0.05 | 7.30±0.60 | 2.74 | 37.53 | 19.66 | 21.22 | 11.29 |
|  | 2021 | 2.04±0.54*** | 1.43±0.29 | 2.76±0.68 | 0.74±0.12 | 7.00±0.78 | 2.54 | 36.25 | 20.44 | 23.73 | 17.07 |
| **CtLwid** | 2020 | 0.30±0.07*** | 0.20±0.04 | 0.40±0.09 | 0.75±0.07 | 3.19±0.28 | 0.98 | 30.79 | 17.22 | 19.84 | 13.93 |
|  | 2021 | 0.31±0.08*** | 0.20±0.04 | 0.41±0.10 | 0.76±0.07 | 3.13±0.29 | 1 | 31.81 | 17.77 | 20.45 | 14.3 |
| **Lwid** | 2020 | 7.14±1.39*** | 1.92±0.37 | 8.10±1.58 | 0.88±0.06 | 11.09±1.07 | 5.17 | 46.62 | 24.1 | 25.67 | 12.49 |
|  | 2021 | 7.22±1.69*** | 3.55±0.71 | 8.99±2.04 | 0.80±0.16 | 10.69±1.30 | 4.96 | 46.4 | 25.14 | 28.06 | 17.62 |
| **Ptillen** | 2020 | 6.90±1.49*** | 3.35±0.57 | 8.57±1.77 | 0.80±0.13 | 11.01±1.17 | 4.85 | 44.07 | 23.85 | 26.59 | 16.63 |
|  | 2021 | 7.64±1.52*** | 1.39±0.32 | 8.34±1.68 | 0.92±0.05 | 10.88±0.91 | 5.45 | 50.14 | 25.42 | 26.55 | 10.84 |
| **LfArea** | 2020 | 539.65±101.31*** | 110.16±19.02 | 594.73±110.81 | 0.91±1.61 | 54.68±7.32 | 45.58 | 83.36 | 42.48 | 44.60 | 19.19 |
|  | 2021 | 565.13±127.80*** | 232.05±46.81 | 681.15±151.21 | 0.83±6.65 | 51.37±10.84 | 44.61 | 86.84 | 46.28 | 50.81 | 29.66 |
| **FBiom** | 2020 | 845.72±185.88*** | 410.03±78.91 | 1050.74±225.34 | 0.80±12.43 | 67.42±13.62 | 53.75 | 79.72 | 43.13 | 48.08 | 30.03 |
|  | 2021 | 1417.92±389.58*** | 1109.70±220.86 | 1972.77±500.01 | 0.72±44.71 | 66.68±21.18 | 65.76 | 98.62 | 56.47 | 66.61 | 49.96 |
| **EDBiom** | 2020 | 109.13±25.59*** | 73.42±12.50 | 145.84±31.84 | 0.75±2.40 | 26.66±5.29 | 18.62 | 69.82 | 39.18 | 45.29 | 32.14 |
|  | 2021 | 238.74±67.23*** | 201.98±40.01 | 339.73±87.24 | 0.70±8.42 | 30.05±8.92 | 26.68 | 88.8 | 51.42 | 61.34 | 47.3 |
| **HI** | 2020 | 0.01±0.00*** | 0.01±0.00 | 0.01±0.00 | 0.76±0.06 | 0.44±0.05 | 0.16 | 37.53 | 20.85 | 23.87 | 16.42 |
|  | 2021 | 0.01±0.00*** | 0.01±0.00 | 0.01±0.00 | 0.74±0.07 | 0.50±0.05 | 0.15 | 30.61 | 17.23 | 19.97 | 14.29 |
| **DM** | 2020 | 1.13±0.31*** | 1.13±0.21 | 1.70±0.41 | 0.67±0.12 | 10.94±0.63 | 1.79 | 16.34 | 9.71 | 11.90 | 9.71 |
|  | 2021 | 0.79±0.28** | 1.02±0.22 | 1.30±0.39 | 0.61±0.16 | 10.33±0.70 | 1.43 | 13.8 | 8.59 | 11.02 | 9.75 |
| **DFlow** | 2020 | 88.98±16.65*** | 12.87±2.89 | 95.41±18.09 | 0.93±0.20 | 70.23±2.84 | 18.76 | 26.72 | 13.43 | 13.91 | 5.11 |
|  | 2021 | 63.3±15.02*** | 30.29±6.68 | 78.44±18.36 | 0.81±1.08 | 49.87±3.83 | 14.72 | 29.52 | 15.95 | 17.76 | 11.04 |

Rep: replicates, StDiam: stem diameter (mm), PHeight: plant height (cm), PBrLeng: primary branch length (cm), NPBr: number of primary branches, CtLleng: central leaflet length (cm), CtLwid: central leaflet width (cm), Lwid: leaf width (cm), Ptillen: petiole length (cm), LfArea: leaf area (cm^2^), FBiom: total fresh biomass per plant (g), EDBiom: edible fresh, biomass per plant (g), HI: harvest index, DM: dry matter content (%), DFlow: days to 50% flowering (days). $\sigma_{e}^{2}$ = residual variance, $\sigma_{G}^{2}$ = genotypic variance, $\sigma_{P}^{2}$ = phenotypic variance, $H^{2}$ = broad-sense heritability, GA: Genetic advance; GAM: genetic advance over mean, GCV: coefficient of genotypic variation; PCV: coefficient of phenotypic variation, ECV: residual coefficient of variation.

***, **, *: significantly different from zero at the 0.001, 0.01, and 0.05 probability level, respectively. ns: not significantly different from zero at the 0.05 level of probability.
